# A Systematic Review of Epstein–Barr Virus Latent Membrane Protein 1 (LMP1) Gene Variants in Nasopharyngeal Carcinoma

**DOI:** 10.3390/pathogens10081057

**Published:** 2021-08-20

**Authors:** Ana Banko, Danijela Miljanovic, Ivana Lazarevic, Andja Cirkovic

**Affiliations:** 1 Institute of Microbiology and Immunology, Faculty of Medicine, University of Belgrade, 11000 Belgrade, Serbia; danijela.karalic@med.bg.ac.rs (D.M.); ivana.lazarevic@med.bg.ac.rs (I.L.); 2 Institute for Medical Statistics and Informatics, Faculty of Medicine, University of Belgrade, 11000 Belgrade, Serbia; andja.cirkovic@med.bg.ac.rs

**Keywords:** EBV, nasopharyngeal carcinoma, LMP1, gene variability, variants, meta-analysis

## Abstract

Nasopharyngeal carcinoma (NPC) is an aggressive tumor with a complex etiology. Although Epstein–Barr virus (EBV) infection is known environmental factor for NPC development, the degree to which EBV naturally infects nasopharyngeal epithelium and the moment when and why the virus actively begins to affect cell transformation remains questionable. The aim of this study was to explore the association between LMP1 gene variability and potential contribution to NPC development. A systematic review was performed through searches of PubMed, Web of Science (WoS) and SCOPUS electronic databases. Additionally, meta-analysis of the difference in the frequency of seven LMP1 gene variants in NPC and control individuals was accomplished. The results from this study give a proof of concept for the association between 30 bp deletion (OR = 3.53, 95% CI = 1.48–8.43) and Xhol loss (OR = 14.17, 95% CI = 4.99–40.20) and NPC susceptibility when comparing biopsies from NPC and healthy individuals. Otherwise, 30 bp deletion from NPC biopsies could not distinguish NPC from EBV-associated non-NPC tumors (OR = 1.74, 95% CI = 0.81–3.75). However, B95-8, China1 and North Carolina variants were uncommon for NPC individuals. Much more efforts remains to be done to verify the biological significance of the differences observed, define so-called “high-risk” EBV variants and make it available for clinical application.

## 1. Introduction

Nasopharyngeal carcinoma (NPC) is a rare but aggressive tumor that originates from the epithelial cells of the retronasal cavity. Although it could be presented with varying degrees of differentiation, the undifferentiated carcinoma of nasopharyngeal type (UCNT, World Health Organization type III) is the most dominant histopathological type in high-risk areas. Unlike most of the world’s population, including Europe and USA where NPC is rare with an incidence below 1 per 100,000 persons per year, in endemic regions of Asia incidence rate is 20–30 per 100,000 persons per year [1]. According to data from 2020, 85.2% of newly registered cases globally belong to the Asian continent [2]. Striking geographic distribution of NPC is the result of the complex etiology of this carcinoma. It has been suggested that both genetic and environmental factors could play a role in the development of NPC. Genetic predisposition is based on HLA (human leukocyte antigen) polymorphisms and chromosomal 3p LOH (loss of heterozygosity), which is supported by finding of NPC clustering in families from diverse populations [3,4]. On the other hand, environmental factors include food common to Southern Chinese cultures, in particular consumption of salted fish, tobacco smoke, alcohol consumption, inhalant and Epstein–Barr virus (EBV) infection [3,5].

The association of NPC and EBV was first discovered by seroepidemiological studies which revealed elevated anti-EBV IgA antibodies in patients with NPC [6,7]. In addition, the level of antibody titers was higher in UCNT patients in comparison to squamous cell carcinoma (SCCs) patients. Additionally, EBV-encoded small RNAs (EBERs), which are expressed in all patterns of EBV latent infection, were detected in UCNTs but not in SCCs [8]. As a key environmental factor of UCNT, EBV infection has been identified as a group 1 carcinogenic agent by the International Agency for Research and Cancer (IARC). EBV latently infects NPC cells and occasionally enters productive lytic infection. However, the degree to which EBV naturally infects nasopharyngeal epithelium and the moment when the virus is acquired by the NPC progenitor population during tumor development remains questionable. The establishment of latent transforming infection based on limited viral protein expression includes activity of latent membrane protein 1 (LMP1), a crucial viral oncogene. It has been shown that LMP1 transforms rodent fibroblast in vitro and induces tumors in nude mice [9,10]. The oncogenic potential of LMP1 is suggested by its high functional similarity to the tumor necrosis factor (TNFR) receptor family members, CD40 and TNFR1 [11]. The effect that LMP1 exerts during NPC pathogenesis includes upregulation of the anti-apoptotic *A20* and *bcl-2* genes, a modulation of the morphology and motility of epithelial cells, a downregulation of metastasis suppressors, promotion of angiogenesis and activation of proinflammatory cytokines [12]. It has been proven that the aggressiveness manifested in metastasis-prone behavior of EBV-positive NPC is particularly associated with the expression of the LMP1 [13].

LMP1 is a 356 amino acid integral membrane protein formed by three domains: a short N-terminal tail (amino acids 1–23); six transmembrane domains (amino acids 24–186); the long cytoplasmic C-terminal tail (amino acids 187–386) with three distinct functional domains or the C-terminal activation regions (CTAR) 1, 2, and 3 [14,15]. Investigation of LMP1 gene variability is particularly interesting since it is significantly heterogeneous with a greater degree of polymorphism than most of the other EBV genes [16]. Seven main LMP1 strains have been defined based on nucleotide sequence variations relative to the wild-type strain B95-8: Alaskan (AL), China1, China2, China3, Mediterranean with (Med+) or without (Med-) deletions, and North Carolina (NC) [17,18]. However, several new strains have been reported in the years following the establishment of the basic classification [19]. It is important to note that LMP1 variants with the 30 bp deletion (30 bp del) correlated with higher transforming ability and lower immunogenic potential of EBV [20]. The similar properties are described in 69 bp deletion (69 bp del) and in the loss of a restriction site in the N-terminal tail, known as XhoI [12,19].

Understanding EBV molecular epidemiology could be crucial in preventing and treating all pathological conditions associated with viral infection. Considering the heterogeneity of the examined populations in previous studies, there are the ambiguous results and the lack of definitive conclusions about the association between LMP1 gene variability and potential contribution to NPC development. The aim of this systematic review and meta-analysis was to explore this problem.

## 2. Results

### 2.1. Systematic Review

**Figure 1:**
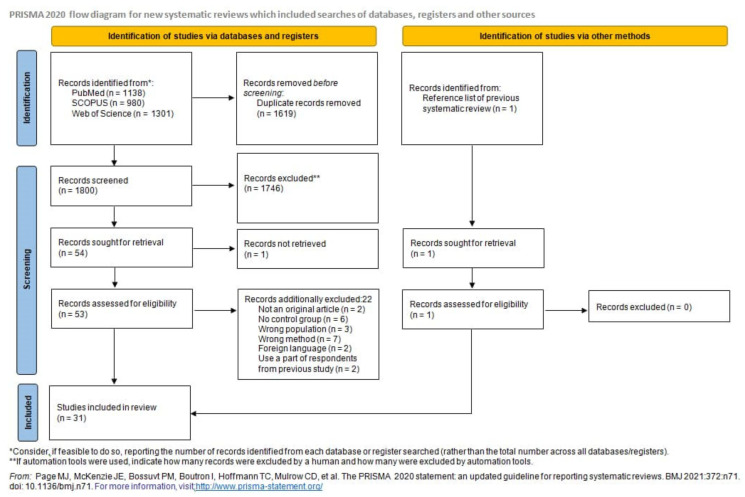
Flow chart.

A total of 3420 potentially eligible articles were found. After duplicates (*n* = 1619) removed, title and abstracts were evaluated for 1801 articles. In total, 1747 articles were excluded because they were not original articles, did not explore NPCs, did not compare NPC and control groups, examined populations other than human (animals, cell lines), explored genes other than LMP1, explored the presence of LMP1 and/or its expression level but not LMP1 variants, or were abstracts. Of the 54 reviewed full text articles, 31 were selected for inclusion in the systematic review. A flow diagram illustrating this selection process is presented in Figure 1.

Characteristics of all 31 publications included in the systematic review are presented in detail in Table 1. They were published between 1992 and 2019, with a total of 3371 participants: 1705 patients with and 1666 without NPC. The number of EBV positive NPC patients was 1595, and there were 1330 EBV positive controls. The minimum sample size of the NPC group was one, and of the control group was three. The maximum size of the NPC group was 154, and the control group was 269. Only 3 studies reported their design (2 case–controls and 1 cross-sectional study). Most studies were from China (9). Six were multicenter studies, while one did not report the country of origin. Other studies were done in Taiwan (2), Russia (3), Spain (1), Malaysia (2), Argentina (1), Morocco (1), Tunis (1), Thailand (1), Serbia (1), Portugal (1), and Iran (1). The majority of all studies (15/31) originated from endemic regions for NPCs. Five out of six multicenter studies were from endemic and non-endemic regions. Five were from non-endemic regions only.

Biopsy epithelial tissue of NPC patients was commonly evaluated (28/31 studies). Other sources for EBV LMP1 variants detection from NPC patients were peripheral blood in 8 studies (whole blood in 3, peripheral blood mononuclear cells in 1, peripheral blood lymphocytes in 1, peripheral blood leukocytes in 1, serum in 1, and plasma in 1 study), throat washing in 5, and xenografts in 1 study. Control groups were very heterogeneous. Most common sources of a control sample were patients with lymphomas, then non-NPC tumors, infectious mononucleosis, nasopharyngeal inflammation, and oral hairy leukoplakia. Three ways of sampling were implemented: biopsy, blood sampling and throat washing.

The age of the participants ranged from 2 to 86 years; however, the predominant age was approximately 50 years. In the NPC group age ranged from 2 to 86 years and in the control group from 5 to 77 years. Regarding the gender of the participants, there was a male predominance with a male/female ratio of 1.87 (in NPC group male/female ratio was 2.5 and in the control group it was 1.2).

Summary of findings from this study is presented in the Supplementary Table (Table S1).

### 2.2. Meta-Analysis of the Association between LMP1 Variants with NPC

Meta-analysis was performed for 7 variants (Xhol loss, 30 bp deletion, 69 bp deletion, B95-8, China1, Mediterranean and North Carolina). First, it was accomplished regardless of the country of patient origin, and then by regions (endemic and non-endemic). Only studies that evaluated and compared the frequency of LMP1 gene variants in human NPC and control groups were taken into account.

#### 2.2.1. Xhol Loss

**Figure 2:**
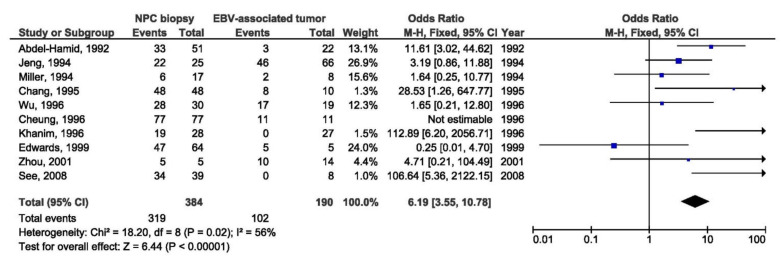
Forest plot of the frequency of the occurrence of Xhol loss in biopsies from NPC and other EBV-associated tumors patients. The confidence interval (CI) was 95%, and the diamond represents the pooled estimate (The blue squares represent point estimation of each study weighted for population size).

**Figure 3:**
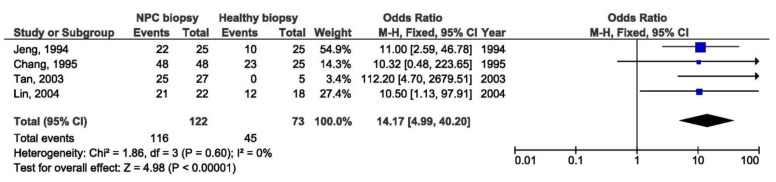
Forest plot of the frequency of the occurrence of Xhol loss in biopsies from NPC and healthy individuals. The confidence interval (CI) was 95%, and the diamond represents the pooled estimate (The blue squares represent point estimation of each study weighted for population size).

**Figure 4:**
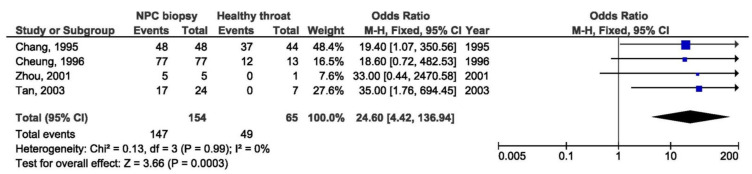
Forest plot of the frequency of the occurrence of Xhol loss in biopsies from NPC and TWs from healthy individuals. The confidence interval (CI) was 95%, and the diamond represents the pooled estimate (The blue squares represent point estimation of each study weighted for population size).

A total of 13 studies were included in the meta-analysis of the association between Xhol loss LMP1 variant and NPC susceptibility. Xhol loss showed strong association with NPC susceptibility between NPC and other EBV-associated tumors biopsies (OR = 6.19, 95% CI = 3.55–10.78, *p* < 0.001) (Figure 2), and even stronger association between NPC and healthy respondents’ biopsies (OR = 14.17, 95% CI = 4.99–40.20, *p* < 0.001) (Figure 3). The presence of Xhol loss enlarged the chance for NPC for 6 times in comparison with other tumors, and 14 times in comparison with healthy tissue. The strongest association of Xhol loss was found in NPC biopsy tissue in comparison with throat washing samples from healthy individuals (OR = 24.60, 95% CI = 4.42–136.74, *p* < 0.001) (Figure 4).

#### 2.2.2. The 30 bp Deletion

A total of 27 studies were included in the meta-analysis of the association between 30 bp deletion LMP1 and NPC susceptibility. Due to the great tissue heterogeneity, the meta-analysis was organized in subgroups according to the type of clinical samples. First, this relationship was evaluated between NPC biopsy samples and other specimens (healthy individual’s biopsies, healthy individual’s throat washings, and EBV-associated non-NPC tumors). Further, it was examined in non-biopsy samples (throat washings and blood) between NPC and healthy controls.

**Figure 5:**
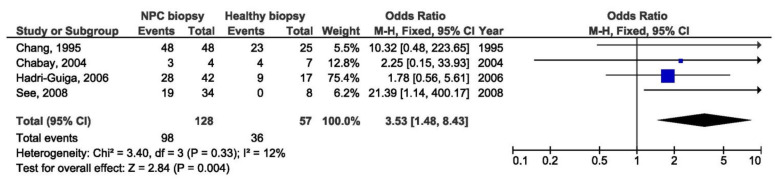
Forest plot of the frequency of the occurrence of 30 bp del in biopsies from NPC and healthy individuals. The confidence interval (CI) was 95%, and the diamond represents the pooled estimate (The blue squares represent point estimation of each study weighted for population size).

**Figure 6:**
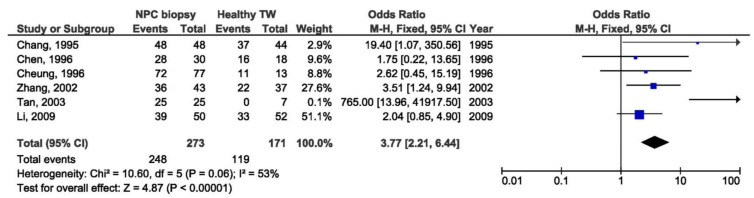
Forest plot of the frequency of the occurrence of 30 bp del in biopsies from NPC and TWs from healthy individuals. The confidence interval (CI) was 95%, and the diamond represents the pooled estimate (The blue squares represent point estimation of each study weighted for population size).

**Figure 7:**
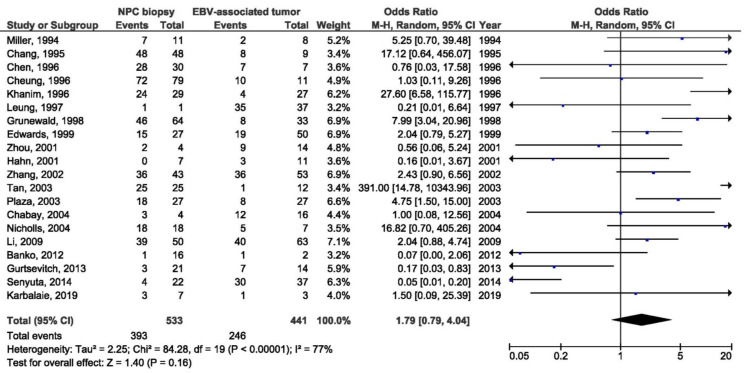
Forest plot of the frequency of the occurrence of 30 bp del in biopsies from NPC and other EBV-associated tumors patients. The confidence interval (CI) was 95%, and the diamond represents the pooled estimate (The blue squares represent point estimation of each study weighted for population size).

**Figure 8:**
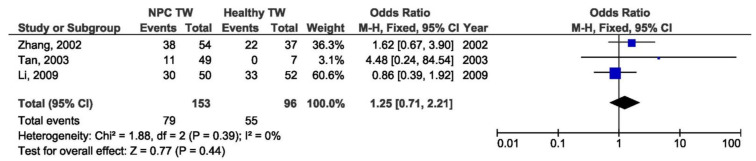
Forest plot of the frequency of the occurrence of 30 bp del in TWs from NPC and healthy individuals. The confidence interval (CI) was 95%, and the diamond represents the pooled estimate (The blue squares represent point estimation of each study weighted for population size).

**Figure 9:**
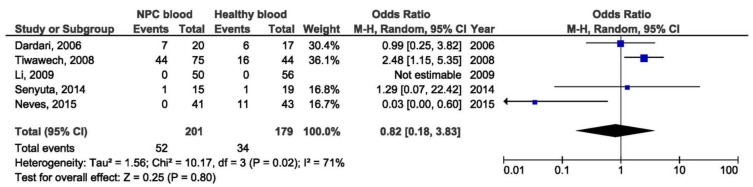
Forest plot of the frequency of the occurrence of 30 bp del in blood from NPC and healthy individuals. The confidence interval (CI) was 95%, and the diamond represents the pooled estimate (The blue squares represent point estimation of each study weighted for population size).

There was a significant association between the 30 bp deletion and NPC susceptibility in two compared groups: NPC biopsy samples and healthy biopsies (OR = 3.53, 95% CI = 1.48–8.43, *p* = 0.004) (Figure 5). The result of the meta-analysis of the difference in the presence of 30 bp deletion LMP1 variant between NPC biopsy samples and throat washings from healthy respondents showed significantly greater frequency of this variant in NPC biopsies (OR = 3.77, 95% CI = 2.21–6.44, *p* < 0.001) (Figure 6). However, when we analyzed the association of the 30 bp deletion LMP1 variant with NPC, when comparing biopsies from NPC and other EBV-associated non-NPC tumors, there was not a significant association (OR = 1.74, 95% CI: 0.81–3.75, *p* = 0.160) (Figure 7). In addition, when non-biopsy NPC samples were compared with the same type of the specimen from healthy controls, no statistically significant difference in the frequency of 30 bp deletion LMP1 variant in throat washings nor in peripheral blood samples was found (OR = 1.25, 95% CI = 0.71–2.21, *p* = 0.440 and OR = 0.82, 95% CI = 0.18–3.83, *p* = 0.800, respectively) (Figure 8 and Figure 9).

#### 2.2.3. The 69 bp Deletion

**Figure 10:**
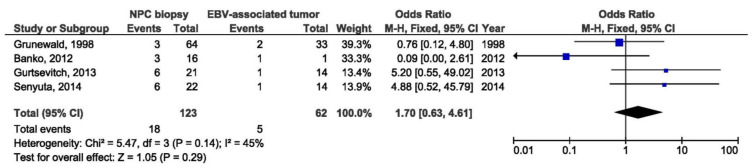
Forest plot of the frequency of the occurrence of 69 bp del in biopsies from NPC and other EBV-associated tumor patients. The confidence interval (CI) was 95%, and the diamond represents the pooled estimate (The blue squares represent point estimation of each study weighted for population size).

**Figure 11:**
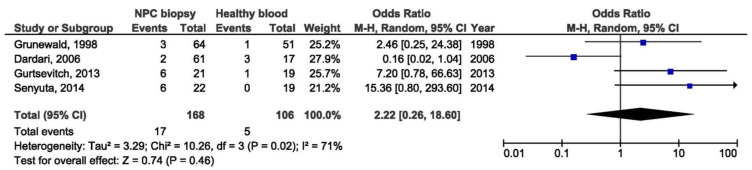
Forest plot of the frequency of the occurrence of 69 bp del in NPC biopsies and blood from healthy individuals. The confidence interval (CI) was 95%, and the diamond represents the pooled estimate (The blue squares represent point estimation of each study weighted for population size).

There were no significant association between 69 bp deletion and NPC susceptibility when comparing biopsies from NPC and other EBV-associated tumors, nor when comparing NPC biopsies and healthy donors blood samples (OR = 1.70, 95% CI = 0.63–4.61, *p* = 0.290, and OR = 2.22, 95% CI = 0.26–18.60, *p* = 0.460) (Figure 10 and Figure 11).

#### 2.2.4. B95-8 Variant

**Figure 12:**
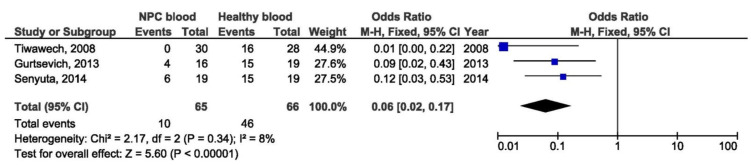
Forest plot of the frequency of the occurrence of B95-8 in blood samples from NPC and healthy individuals. The confidence interval (CI) was 95%, and the diamond represents the pooled estimate (The blue squares represent point estimation of each study weighted for population size).

**Figure 13:**
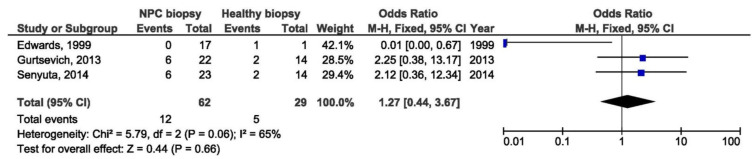
Forest plot of the frequency of the occurrence of B95-8 loss in biopsies from NPC and healthy individuals. The confidence interval (CI) was 95%, and the diamond represents the pooled estimate (The blue squares represent point estimation of each study weighted for population size).

**Figure 14:**
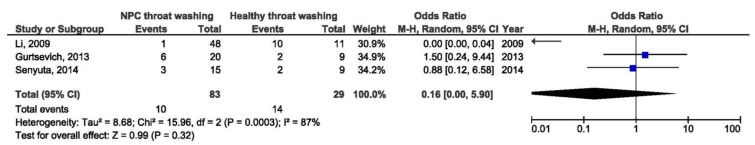
Forest plot of the frequency of the occurrence of B95-8 in TWs from NPC and healthy individuals. The confidence interval (CI) was 95%, and the diamond represents the pooled estimate (The blue squares represent point estimation of each study weighted for population size).

B95-8 variant in blood samples from NPC patients was a protective factor for NPCs when comparing with blood specimens from healthy individuals (OR = 0.06, 95% CI = 0,02–0,17, *p* < 0.001) (Figure 12). In addition, there was no significant association between B95-8 variant and NPC susceptibility when comparing biopsies from NPC and other EBV-associated tumors, but also when comparing throat washing samples from NPC and healthy individuals (OR = 1.27, 95% CI = 0.44–3.67, *p* = 0.660 and OR = 0.16, 95% CI = 0.00–5.90, *p* = 0.320, respectively) (Figure 13 and Figure 14).

#### 2.2.5. China1 Variant

**Figure 15:**
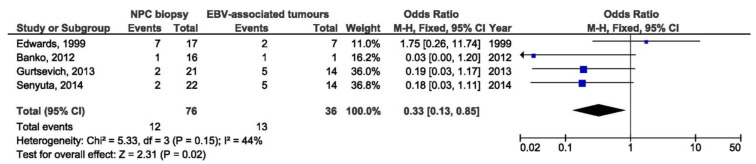
Forest plot of the frequency of the occurrence of China1 in biopsies from NPC and other EBV-associated tumors patients. The confidence interval (CI) was 95%, and the diamond represents the pooled estimate (The blue squares represent point estimation of each study weighted for population size).

**Figure 16:**
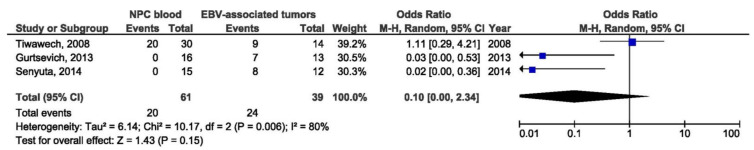
Forest plot of the frequency of the occurrence of China1 in blood samples from NPC and other EBV-associated tumors patients. The confidence interval (CI) was 95%, and the diamond represents the pooled estimate (The blue squares represent point estimation of each study weighted for population size).

**Figure 17:**
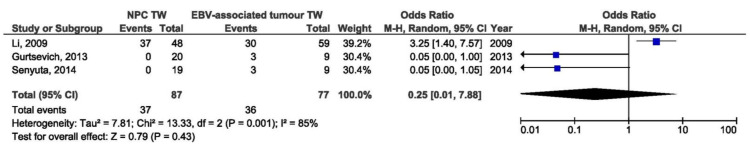
Forest plot of the frequency of the occurrence of China1 in TWs from NPC and other EBV-associated tumors patients. The confidence interval (CI) was 95%, and the diamond represents the pooled estimate (The blue squares represent point estimation of each study weighted for population size).

A total of six studies were included in the meta-analysis of the association between China1 LMP1 variant and NPC susceptibility. There was a significant inverse association between China1 LMP1 variant and NPC susceptibility when comparing biopsies from NPC and EBV-associated non-NPC tumors (OR = 0.16, 95% CI = 0.05–0,52, *p* = 0.003) (Figure 15). Otherwise, there was no significant association between China1 LMP1 variant and NPC when comparing blood samples from NPC and EBV-associated non-NPC tumors (OR = 0.10, 95% CI = 0.00–2.34, *p* = 0.150) (Figure 16), as well as when comparing throat washings from NPC and EBV-associated non-NPC tumors (OR = 0.25, 95% CI = 0.01–7.88, *p* = 0.430) (Figure 17).

#### 2.2.6. Mediterranean (Med) Variant

**Figure 18:**
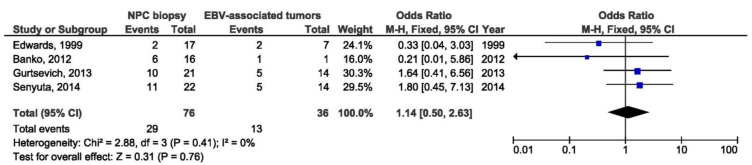
Forest plot of the frequency of the occurrence of Med in biopsies from NPC and other EBV-associated tumors patients. The confidence interval (CI) was 95%, and the diamond represents the pooled estimate (The blue squares represent point estimation of each study weighted for population size).

**Figure 19:**
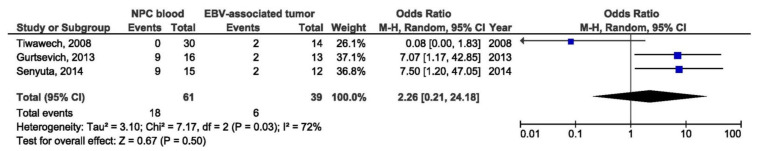
Forest plot of the frequency of the occurrence of Med in blood samples from NPC and other EBV-associated tumors patients. The confidence interval (CI) was 95%, and the diamond represents the pooled estimate (The blue squares represent point estimation of each study weighted for population size).

**Figure 20:**
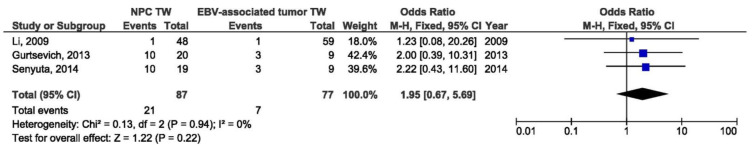
Forest plot of the frequency of the occurrence of Med in TWs from NPC and other EBV-associated tumors patients. The confidence interval (CI) was 95%, and the diamond represents the pooled estimate (The blue squares represent point estimation of each study weighted for population size).

A total of six studies were included in the meta-analysis of the association between Med LMP1 variant and NPC susceptibility. There was no association between Med LMP1 variant and NPC when comparing biopsy samples from NPC and EBV-associated non-NPC tumors (OR = 1.14, 95% CI = 0.50–2.63, *p* = 0.760) (Figure 18). Additionally, no association between Med LMP1 variant and NPC was found when comparing blood samples from NPC and EBV-associated non-NPC tumors (OR = 2.26, 95% CI = 0.21–24.18, *p* = 0.500) (Figure 19). Med LMP1 variant was not in a relation with NPC as well when comparing throat washings from NPC and EBV-associated non-NPC tumors (OR = 1.95, 95% CI = 0.67–5.69, *p* = 0.220) (Figure 20).

#### 2.2.7. North Carolina (NC) Variant

**Figure 21:**
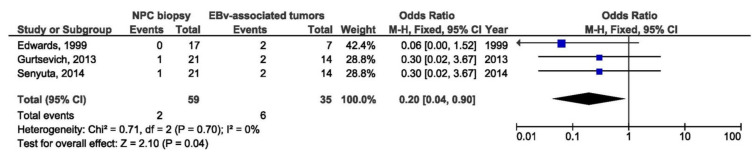
Forest plot of the frequency of the occurrence of NC in biopsies from NPC and other EBV-associated tumors patients. The confidence interval (CI) was 95%, and the diamond represents the pooled estimate (The blue squares represent point estimation of each study weighted for population size).

A total of three studies were included in the meta-analysis of the association between North Carolina LMP1 variant and NPC susceptibility. NC LMP1 variant in NPC biopsy samples was a protective factor when comparing with other EBV-associated tumor biopsies (OR = 0.20, 95% CI = 0.04–0.90, *p* = 0.040) (Figure 21).

### 2.3. The Association between LMP1 Variants and NPC Susceptibility by Regions: Endemic and Non-Endemic

#### 2.3.1. The 30 bp Deletion

**Figure 22:**
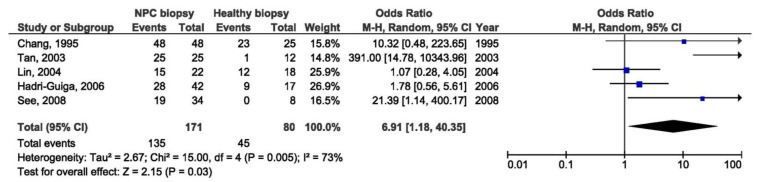
Forest plot of the frequency of the occurrence of 30 bp del in biopsies from NPC and healthy individuals in endemic regions. The confidence interval (CI) was 95%, and the diamond represents the pooled estimate (The blue squares represent point estimation of each study weighted for population size).

**Figure 23:**
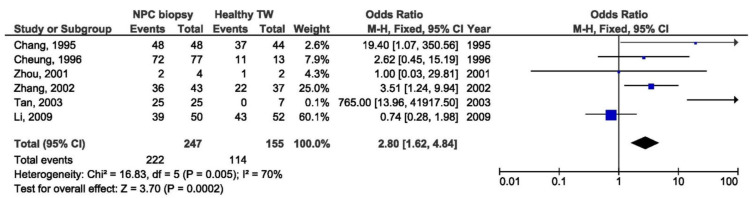
Forest plot of the frequency of the occurrence of 30 bp del in NPC biopsies and TWs from healthy individuals in endemic regions. The confidence interval (CI) was 95%, and the diamond represents the pooled estimate (The blue squares represent point estimation of each study weighted for population size).

**Figure 24:**
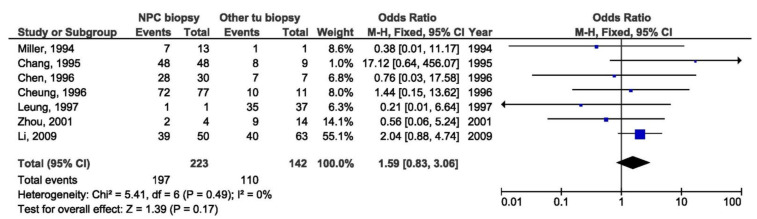
Forest plot of the frequency of the occurrence of 30 bp del in biopsies from NPC and other EBV-associated tumors patients in endemic regions. The confidence interval (CI) was 95%, and the diamond represents the pooled estimate (The blue squares represent point estimation of each study weighted for population size).

Further meta-analysis was done by regions: endemic and non-endemic. There was a significant association between 30 bp del LMP1 and the NPC susceptibility in the studies conducted in endemic regions when comparing biopsies from NPC and healthy individuals (OR = 6.91, 95% CI = 1.18–40.35, *p* = 0.030) (Figure 22), but also when comparing NPC biopsy and throat washings from healthy individuals (OR = 2.80, 95% CI = 1.62–4.84, *p* < 0.001) (Figure 23). Otherwise, there was no significant difference in the prevalence of 30 bp del in studies from endemic regions when comparing biopsies from NPC and EBV-associated non-NPC tumors (OR = 1.59, 95% CI = 0.83–3.06, *p* = 0.170) (Figure 24).

**Figure 25:**
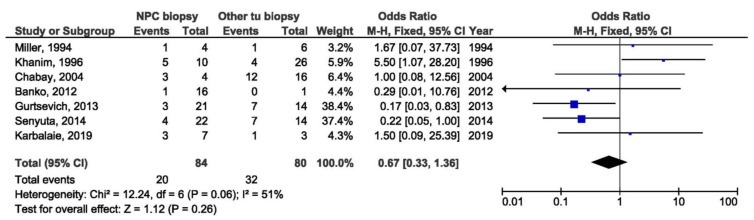
Forest plot of the frequency of the occurrence of 30 bp del in biopsies from NPC and other EBV-associated tumors patients in non-endemic regions. The confidence interval (CI) was 95%, and the diamond represents the pooled estimate (The blue squares represent point estimation of each study weighted for population size).

Additionally, there was no association between 30 bp del and NPC susceptibility in studies from non-endemic regions when comparing biopsies from NPC and EBV-associated non-NPC tumors patients (OR = 0.67, 95% CI = 0.33–1.36, p = 0.260) (Figure 25).

#### 2.3.2. Xhol Loss

**Figure 26:**
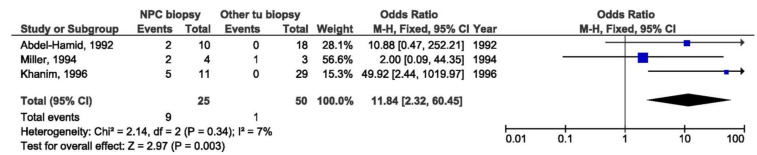
Forest plot of the frequency of the occurrence of Xhol loss in biopsies from NPC and other EBV-associated tumors patients in non-endemic regions. The confidence interval (CI) was 95%, and the diamond represents the pooled estimate (The blue squares represent point estimation of each study weighted for population size).

**Figure 27:**
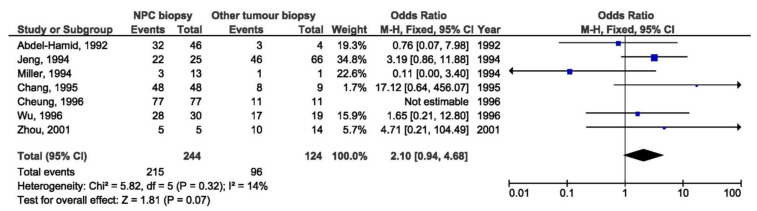
Forest plot of the frequency of the occurrence of Xhol loss in biopsies from NPC and other EBV-associated tumors patients in endemic regions. The confidence interval (CI) was 95%, and the diamond represents the pooled estimate (The blue squares represent point estimation of each study weighted for population size).

It was shown that Xhol loss was in statistically significant relation with NPC when comparing biopsies from NPC and EBV-associated non-NPC patients from non-endemic regions (OR = 11.84, 95% CI = 2.32–60.45, *p* = 0.003) (Figure 26), but there was no association between Xhol loss and the NPC susceptibility when comparing biopsies from NPC and EBV-associated non-NPC patients from endemic regions (OR = 2.10, 95% CI = 0.94–4.68, *p* = 0.070) (Figure 27).

## 3. Discussion

This study provided a comprehensive systematic review of LMP1 gene variability, not only between NPC and non-NPC participants in general, but a much more homogeneous and detailed comparison. For the first time, a comparative analysis of LMP1 gene variability included seven variants: Xhol loss, 30 bp and 69 bp deletions, B95-8, China1, Mediterranean, and North Carolina variants, which were presented in different tumor-altered and healthy tissues. Finally, a comparison of the frequency of LMP1 gene variants was also made in relation to geographical origin of the clinical sample.

The course of the NPC pathogenesis is influenced by several factors, among which are genetic susceptibility, environmental and viral factors. As far as viral factors are concerned, EBV and Human papilloma virus (HPV) infections are the most investigated and mentioned in this context. Only recently, the results of meta-analysis designed to establish the relationship between viral combinations in NPC stratified according to histological type have been published [50]. While keratinizing NPC subtype (WHO type I) is found mainly in non-endemic areas, in endemic areas with a high incidence of disease, the majority of cases are non-keratinizing subtypes (WHO types II and III) [51]. Even though keratinizing NPC subtype is often HPV positive, no clear association has been established yet [52,53]. On the other hand, non-keratinizing NPC subtypes are almost always EBV positive and proven to be associated with EBV infection [54]. In addition, EBV has been linked to a wide range of other lymphoid and epithelial cell malignancies such as posttransplant lymphoproliferative disease (PTLD), Burkitt’s, Hodgkin’s and nasal natural killer/T-cell lymphomas, and gastric adenocarcinoma [54].

Several different viral oncogenes such as LMP1, LMP2, and EBNA1 play an important role in the pathogenesis of EBV-associated tumors. The majority of the studies, concerning EBV genetic variability, have focused attention and investigation on LMP1 gene polymorphisms, which is at the same time the most important EBV oncogene but also the most variable gene. Having this in mind, a previous meta-analysis determined the impact of 30 bp deletion and Xhol loss on the risk for NPC development [12]. The authors found that there was a positive association between 30 bp deletion and Xhol loss and NPC susceptibility, but without confirmation that these two LMP1 variants could be considered and used as specific markers of the NPC. Actually, 30 bp deletion and Xhol loss were also detected in control groups and in the other EBV-associated cancers. Our study confirmed the previous data, but with the significantly greater association. Particularly, 30 bp deletion was found 3.5 or 3.8 times more frequently in the NPC biopsies than in the biopsies or TWs from healthy people, respectively. The association between Xhol loss and the NPC susceptibility was much stronger because the presence of Xhol loss in the NPC biopsies was even 14 times higher than in healthy tissue, and as many as 24.6 times than in TWs from healthy individuals. Those frequencies are significantly higher than 8.5 which was reported by previous meta-analysis although some literature data reported the complete absence of Xhol loss in the healthy population [12,26].

The systematic classification of the clinical samples’ origin and their comparative analysis improved the validity of results obtained in this study. Therefore, it becomes clearer that the specificities of the viral genome are primarily related to tumor-altered tissue, not only NPC but also tissues of the other EBV-related tumors which were included and analyzed in this meta-analysis. Thus, from our results, it could be seen that the association between the LMP1 deleted variant and NPC was seen in viral genome which was localized in the tumor-altered tissue itself, and that it is absent when the frequency of 30 bp deletion is compared with any other type of clinical sample. These findings support published data about the time specific and determined role of EBV oncogenic activity in early phases of NPC development [55,56]. Actually, it is very striking that the viral genome is present and transcriptionally active in every tumor cell, while the healthy nasopharyngeal tissue could not be normally infected with EBV and hardly be able to be a reservoir of latent EBV infection [54]. One of the theories about establishing stable latent EBV infection in pre-neoplastic changes in nasopharyngeal epithelium is based on enforced over-expression of cyclin D in those cells [57]. When the reports about the direct influence of 30 bp deleted LMP1 variant of EBV on the aggressiveness of the carcinogenesis is added to previously mentioned knowledge, it could be assumed that functional differences between LMP1 variants significantly affect the triggering of the cell transformation [58]. It is therefore important to note that the presence of other deletions, such as 69 bp, have no significance in relation to NPC or any other EBV-related tumor development which is also confirmed by this meta-analysis [59].

Extensive research of LMP1 gene heterogeneity has so far defined more than seven different strains. The functional differences between the LMP1 of epithelial cancer-derived EBV, which belongs to China1, and lymphoid-derived EBV, which belongs to B95-8, are well described [60]. However, except the fact that nomenclature of these variants reflects their geographic origin or the location from where they were found and dominate in frequency, none of the mentioned variants could be associated with nasopharyngeal carcinoma in this or previous studies. On the other hand, it is interesting that some of the variants could still be considered as a protective factor for NPC. Thus, the results of this meta-analysis showed that China1 and NC could be considered as protective factors for NPC when comparing to other EBV-associated tumors, and B95-8 variant as a protective factor for NPC when comparing to healthy people. The principle of existence of the so-called “eliminated variants” is described by negative immune selection against the presence of other variants within the tumor [61]. For example, the absence of the North Carolina variant within NPC was explained by the inability of the NC to inhibit T-cell proliferation and natural killer cytotoxicity because of unique mutations in the region of LMP1 gene responsible for immunosuppressive function [61].

Meta-analysis by regions reveals that the presence of 30 bp deletion in the NPC biopsies was almost seven times higher than in healthy tissue in endemic regions. As it has long been postulated that distinct EBV strains are predominant in areas with a high incidence of the NPC, and that EBV strain variation contributes to the endemic nature of the NPC, 30 bp deletion and Xhol loss in these areas could serve as prognostic markers for tumor development [21,62]. This includes the NPC or other EBV-associated tumors since in this study no difference has been shown in association between 30 bp deletion or Xhol loss and either the NPC susceptibility or other EBV-associated tumors susceptibility according to tissue biopsy findings. On the other hand, in non-endemic regions of the NPC, the presence of Xhol loss in NPC biopsies was almost 12 times higher than in the other EBV-associated tumor biopsies. Whether these results from non-endemic regions covered mainly keratinizing subtypes of the NPC with better prognosis remains to be further examined and analyzed in order to potentially define “less oncogenic” and “more oncogenic” EBV variants [54].

As the EBV genetic variability led to the increasing interest of possible disease-specific associations with EBV variants, but still too diverse to single out the individual risk factors, new high-throughput sequencing technologies marked a new era of EBV sequencing. Therefore, a recent explosive increase in EBV whole genome sequences could open the window of new opportunities in EBV genome research, and lead to identification of high-risk groups of people who are predisposed to the NPC and impact on the early diagnosis of the NPC [63]. In addition, it could also determine a new path of EBV taxonomy and classification.

This is not the first, but it is a comprehensive meta-analysis of the association between LMP1 variants and the NPC susceptibility. Regardless of its advantages, some limitations were recognized. First, only articles in English were included (there were 67 articles in other languages). Second, abstracts were not taken into account. The first and the second reason might lead to making a selection bias and losing the data. In order to examine publication bias, Funnel plots were performed, and they indicated no publication bias. Third, there was great heterogeneity in types of clinical samples, so it was chosen to do meta-analysis in subgroups, according to the types of clinical samples taken from NPC and control groups to make more homogeneous conclusions. Fourth, the number of studies in each meta-analysis was different. Some of them, with smaller number of included studies (three) had smaller statistical power than those with greater number of included articles. Fifth, because of the scarcity of data with potential influence on the association between LMP1 variants and NPC susceptibility, such as age and gender, we were not able to perform the meta-regression analysis in order to control the estimated effect size (OR) and avoid underestimation and overestimation in these situations. Sixth, regardless of the duration of performing studies on the association between LMP1 variants and NPC (from 1973 to today) there was not enough survival data in order to perform meta-analysis of hazards.

In summary, the results from this study give a proof of concept for the association between two LMP1 variants (30 bp deletion and Xhol loss) and the NPC susceptibility when comparing biopsies from the NPC and healthy individuals. Moreover, this association was also found with other EBV-associated tumors. Otherwise, 30 bp deletion from NPC biopsies could not distinguish NPC from EBV-associated non-NPC tumors. On the other hand, B95-8, China1 and North Carolina variants were uncommon for NPC individuals. Although it is clear that not only EBV LMP1 gene variability but also many other non-viral factors are involved in the etiopathogenesis of the NPC, the importance of identification of so-called “high-risk“ EBV variants could be crucial in defining relative variant tropism, designing targeted antiviral candidates or even make progress toward an EBV vaccine. Over the past decades, numerous efforts have been made, but much more work remains to be done to verify the biological significance of the differences observed and make it available for clinical application.

## 4. Materials and Methods

This systematic review was performed in accordance with the PRISMA protocol (Reporting Items for Systematic Reviews and Meta-Analyses) and MOOSE guidelines for observational studies after systematic review registration at PROSPERO (Systematic review registration statement is available at https://osf.io/znrwj/) [64,65].

### 4.1. Study Selection

Publications were screened for inclusion in the systematic review in two phases, and all disagreements were resolved by discussion at each stage with inclusion of a third reviewer. We included studies that detected LMP1 gene variants in patients with nasopharyngeal carcinoma and any other group for comparison. Studies were eligible for inclusion if LMP1 gene variants were detected in both groups. Studies were excluded if they (i) investigated other outcomes, (ii) did not make comparisons between patients with nasopharyngeal carcinoma and control groups, (iii) examined other populations (animal, cell lines), (iv) assessed other genes (LMP2, EBNA-1, etc.), (v) did not assess LMP1 variants, but its function or expression, (v) were abstracts, or (vi) were not original articles.

### 4.2. Database Search

Two biostatisticians with expertise in conducting systematic reviews and meta-analyses (AC, DM) developed the search strategy. A systematic review of peer-reviewed publications was performed through searches of PubMed, Web of Science (WoS) and SCOPUS electronic databases until 28 April 2021. Search queries differed according to the database. Keywords for the PubMed search were (Nasopharyngeal carcinoma or Nasopharyngeal neoplasm*) and (Epstein Barr Virus or Epstein-Barr Virus or EBV or Burkitt's lymphoma virus or Herpesvirus 4, human or HHV4) and (TNF receptor associated factor 2 or LMP1-Associated Protein 1 or LMP1 or variant or mutation or deletion or polymorphism); for WoS: ALL = (Nasopharyngeal carcinoma OR Nasopharyngeal neoplasm*) AND ALL = (Epstein Barr Virus OR Epstein-Barr Virus OR EBV OR Burkitt's lymphoma virus OR Human herpesvirus 4 OR HHV4) AND ALL = (TNF Recept associated factor 2 OR LMP1-Associated Protein 1 OR LMP1 or variant or mutation or deletion or polymorphism), and for SCOPUS: (TITLE-ABS-KEY (“Nasopharyngeal carcinoma”) OR TITLE-ABS-KEY (“Nasopharyngeal neoplasm*”) AND TITLE-ABS-KEY (“Epstein Barr Virus”) OR TITLE-ABS-KEY (“Epstein-Barr Virus”) OR TITLE-ABS-KEY (“EBV”) OR TITLE-ABS-KEY (“BURKITT’S LYMPHOMA VIRUS”) OR TITLE-ABS-KEY (“Human herpesvirus 4”) OR TITLE-ABS-KEY (“HHV4”) AND TITLE-ABS-KEY (“TNF RECEPT ASSOCIATED FACTOR 2”) OR TITLE-ABS-KEY (“LMP1-Associated Protein 1”) OR TITLE-ABS-KEY (“LMP1”) OR TITLE-ABS-KEY (“variant”) OR TITLE-ABS-KEY (“mutation”) OR TITLE-ABS-KEY (“deletion”) OR TITLE-ABS-KEY (“polymorphism”). Only publications in English were taken into account. In addition, reference lists of articles identified through electronic retrieval were manually searched, as well as relevant reviews and editorials. Experts in the field were contacted to identify other potentially relevant articles. Authors of relevant articles were contacted to obtain missing data.

### 4.3. Article Screening and Selection

Two reviewers (A.C., D.M.) independently evaluated the eligibility of all titles and abstracts. Studies were included in the full text screening if either reviewer identified the study as being potentially eligible, or if the abstract and title did not include sufficient information. Studies were eligible for full text screening if they included detection of LMP1 gene variants in patients with nasopharyngeal carcinoma and control group. The same reviewers independently performed full text screening to select articles for inclusion according to the criteria listed under Inclusion and Exclusion Criteria. Disagreements were resolved by consensus (A.C., D.M.) or arbitration (A.B., I.L.).

### 4.4. Data Abstraction and Quality Assessment

Two reviewers independently abstracted the following data: author(s), country of research, year of publication, study design, sample size, study population, EBV positivity, type of LMP1 gene variant, method for detection of LMP1 gene variant. Each reviewer independently evaluated the quality of selected manuscripts using an adapted version of the Newcastle–Ottawa tool for observational studies [66]. Reviewers used a standardized previously defined LMP1 variant protocol when selecting and abstracting data. All detailed information about reasons for inclusion/exclusion and quality assessment are available at https://osf.io/znrwj/.

### 4.5. Statistical Analysis

The primary outcome was the frequency of LMP1 gene variants. As the outcome is dichotomous and the sample size varies Mantel–Haenszel method was used as a measure of effect size to examine the differences in ratio of a specific LMP1 gene variant in evaluated study groups from all primary articles. Mantel–Haenszel method is a fixed-effect meta-analysis method that uses a different weighting scheme that depends on which effect measure is being used. Heterogeneity was assessed using the Chi-square Q test and I2 statistic. I2 presents the inconsistency between the study results and quantifies the proportion of observed dispersion that is real, i.e., due to between-study differences and not due to random error. The categorization of heterogeneity was based on the Cochrane Handbook [67] and states that I2 < 30%, 30% to 60% or >60%, corresponds to low, moderate and high heterogeneity, respectively. Forest plots were constructed for each analysis showing the OR (box), 95% confidence interval (lines), and weight (size of box) for each trial. The overall effect size was represented by a diamond. Due to great heterogeneity of control groups, meta-analysis was performed as a subgroup analysis. According to the available data for 30 bp deletion LMP1 variant we performed the following comparisons: NPC biopsy vs. Other EBV-associated tumors biopsy, NPC biopsy vs. Healthy individual’s biopsy, NPC biopsy vs. Throat washing of healthy individuals, NPC blood vs. Healthy individual blood, NPC throat washings vs. Healthy individual’s throat washings. For the Xhol loss variant the following comparisons were performed: NPC biopsy vs. EBV-associated non-NPC tumors, NPC biopsy vs. Healthy individual’s biopsy, and NPC biopsy vs. Healthy individual’s throat washings. It was possible to perform three comparisons for China1 variant: NPC biopsy vs. EBV-associated non-NPC tumors biopsy, NPC blood vs. Healthy individual’s blood, and NPC throat washings vs. EBV-associated non-NPC throat washings. For North Carolina (NC) variant only one comparison was possible—NPC biopsy vs. EBV-associated non-NPC tumors biopsy. Three comparisons were done for B95-8 variant: NPC biopsy vs. EBV-associated non-NPC tumors biopsy, NPC blood vs. Healthy individual’s blood, and NPC throat washings vs. Healthy individual’s throat washings. It was possible to perform three comparisons for Med variant: NPC biopsy vs. EBV-associated non-NPC tumors biopsy, NPC blood vs. Healthy individual’s blood, and NPC throat washings vs. EBV-associated non-NPC throat washings. Additionally, for 69 bp deletion two comparisons were performed: NPC biopsy vs. EBV-associated non-NPC tumors and NPC biopsy vs. Healthy individuals blood samples. Additionally, meta-analysis within endemic and non-endemic regions was performed. Southern China and Hong Kong, Southeast Asia, North Africa and the Arctic are considered endemic regions (Wu, 2018). Publication bias was evaluated by Funnel plot for every outcome (available at https://osf.io/znrwj/.). A *p* value <0.05 was considered to be statistically significant. Analyses were performed using Review Manager Version 5.4 [68].

## Figures and Tables

**Table 1:** Characteristics of studies included in the systematic review.

Author, Year	Country	NPC Cases				Controls			LMP1 Gene Variants in NPC Group													LMP1 Gene Variants in Control Group	Method
		Study Design	*n*	EBV+	Sample Characteristics	n	EBV+	Sample Characteristics	30 bp Del	Xhol-Loss	Ncol Loss	69 bp Del	27 bp Del	B95-8	China1	Med	China2	China3	Alaskan	NC	Other		
Abdel- Hamid, 1992 [21]	Multicenter (Southern China, Malaysia, continental United States, Alaska, Egypt, and equatorial Africa)	NR	56	56	Biopsy	22	22	Total		33												3	Hybridization
						2	2	PGC														2	
						13	13	BL														0	
						6	6	Non-HL														1	
						1	1	HL														0	
Jeng, 1994 [22]	Taiwan	NR	32	25	Biopsy	197	103	Total		22												63	Sequencing
						53	25	Healthy laboratory worker volunteers														10	
						26	12	Patients with tonsillitis and pharyngitis														7	
						118	66	Other head and neck carcinoma														46	
Miller, 1994 [17]	Multicenter (China, Malaysa, USA, Alaska, Mediteran)	NR	17	17	Biopsy	8	8	Total	7	6												2 Xhol loss 2 30 bp del	PCR and sequencing
						2	2	Posttransplant lymphoma														1 Xhol loss 0 30 bp del	
						1	1	BL														0 Xhol loss 0 30 bp del	
						4	4	OHL														0 Xhol loss 1 30 bp del	
						1	1	PGC														1 Xhol loss 1 30 bp del	
Chang, 1995 [23]	Taiwan	NR	48	48	Biopsy	128	78	Total	48	48	0											68 Xhol loss 10 Ncol loss 68 30 bp del	PCR, restriction-enzyme digestion, sequencing
						40	25	Normal nasopharynx tissues														23 Xhol loss. 2 Ncol loss 23 30 bp del	
						78	44	TWs														37 Xhol loss. 7 Ncol loss 37 30 bp del	
						7	7	TCL														6 Xhol loss 1 Ncol loss 6 30 bp del	
						2	1	HL														1 Xhol loss 0 Ncol loss 1 30 bp del	
						1	1	BCL														1 Xhol loss 0 Ncol loss 1 30 bp del	
Chen, 1996 [24]	Not reported	NR	40	40	Biopsy	56	56	Total	28													53 30 bp del	PCR and sequencing
						10	10	Non-NPC biopsy samples														7 30 bp del	
						24	24	TWs														16 30 bp del	
						22	22	PB lymphocytes														20 30 bp del	
Cheung, 1996 [25]	China	NR	77	77	Biopsy and xenografts	24	24	Total	72	77												21 30 bp del 24 Xhol loss	
						11	11	Biopsy samples of gastric carcinomas and gastric lymphomas														10 30 bp del 11 Xhol loss	PCR-RFLP
						13	13	TWs of healthy individuals defined as disease-free close relatives of existing NPC patients														11 30 bp del 13 Xhol loss	
Khanim, 1996 [26]	Multicenter (UK, Taiwan, China, Europe, Africa, New Guinea)	NR	30	30	Biopsy	37	35	Total	24	19												8 30 bp del 0 Xhol loss	PCR, RFLP, sequencing
						4	4	Gastric adenocarcinoma biopsies														2 30 bp del 0 Xhol loss	
						25	23	HL														2 30 bp del 0 Xhol loss	
						8	8	IM														4 30 bp del 0 Xhol loss	
Wu, 1996 [27]	China	NR	30	30	Biopsy	22	22	Total		28												20 Xhol loss	PCR and sequencing
						19	19	nasal and extranasal TCL														17 Xhol loss	
						3	3	IM														3 Xhol loss	
Leung, 1997 [28]	China	NR	1	1	EBV+ metastatic NPC in the lung biopsy	92	92	Total	1													61	PCR and sequencing
						55	52	Resected tonsils from patients with chronic tonsillitis														26	
						6	6	LELC-LG														5	
						10	10	LELCSG														10	
						5	5	SNCAs														4	
						16	16	GACAs														16	
Grunewald, 1998 [29]	Multicenter Europe (Italy and France), North Africa, Asia, Oceania	NR	64	64	Biopsy	94	94	Total	46		9	3										44 30 bp del 4 69 bp del 34 Ncol loss	Sequencing
						12	12	Post-transplant BCL														4 30 bp del 0 69 bp del 5 Ncol loss	
						15	15	Lymphomas of HIV patients (BL, BCL, primary brain lymphoma)														3 30 bp del 0 69 bp del 9 Ncol loss	
						10	10	Lymphocytes from patients with IM														3 30 bp del 1 69 bp del 5 Ncol loss	
						6	6	OHL														1 30 bp del 2 69 bp del 1 Ncol loss	
						51	51	PB-cell pellets from HBD														33 30 bp del 1 69 bp del 14 Ncol loss	
Edwards, 1999 [18]	Multicenter (China, Malaysia, Taiwan, Mediterranean, USA, Alaska)	NR	64 N terminus + 27 C terminus + 17 Full lenth LMP1	108	Biopsy	10 N terminus + 50 C terminus + 7 full lenth LMP1	67	IM, PGC, BL, OHL, Post/transplant lymphoma	15	47				0	7	2	6	1	1	0		5 Xhol loss 19 30 bp del 2 China1 0 China2 0 China3 0 Med+ 2 Med- 1 Alaskan 2 NC	Sequencing
Hahn, 2001 [30]	Russia	NR	7	7	Biopsy	11	11	NPC-like tumor of the parotid gland, healthy carriers’ PB lymphocytes	0													3	Sequencing
Zhou, 2001 [31]	China	NR	6	6	Biopsy	94	30 sequenced	Total	2	5												10 30 bp del 11 Xhol loss	Sekvencing
						71	71 64 LMP1 positive (14 N terminus + 12 C terminus for sequencing)	HD biopsy														8 30 bp del 10 Xhol loss	
						21	21 (2 N terminus + 2 C terminus for sequencing)	TWs from healthy Chinese														1 30 bp del 1 Xhol loss	
						2	2 (only 2 C terminus sequencing)	Chinese nasal TNKLs														1 30 bp del 0 Xhol loss	
Zhang, 2002 [32]	China	NR	154	97	Total	209	90	Total	74													58	Sequencing
			47	43 LMP1+	Biopsy	106	53 LMP1+	TWs from breast, lung–non head and neck carcinoma stomach, colon, and ovary carcinoma patients	36													36	
			107	54 LMP1+	TW	103	37 LMP1+	TWs from healthy donors	38													22	
Lin, 2003 [33]	China	NR	63	63	Biopsy	10	10	PBMCs from healthy donors		54												0	PCR with XhoI digestion and sequencing
Plaza, 2003 [34]	Spain	NR	27	27	Biopsy	27	27	EBV-related IM	18													8	PCR
Tan, 2003 [35]	Malaysia	NR	150	74 LMP+ 48 Xhol	Total	26	19 LMP+ 5 Xhol	Total														1 30 bp del 0 Xhol loss	PCR and restriction enzyme digestion
			120	49 LMP+ 21 Xhol	TW	14	7 LMP+ 5 Xhol	TWs from healthy individuals	11	17												0 30 bp del 0 Xhol loss	
			30	25 LMP+ 27 Xhol	Biopsy	12	12 LMP+	Biopsy from controls with clinical symptoms indicative of nasopharyngeal carcinoma but whose postnasal space biopsies were confirmed as histologically normal	25	25												1 30 bp del 0 Xhol loss	
Chabay, 2004 [36]	Argentina	NR	4	4	Biopsy	27	27	Total														16	PCR and sequencing
						11	11	Non-neoplastic controls	3													4	
						16	16	Non-NPC EBV-related malignancies (HL and non-HL)														12	
Lin, 2004 [37]	Multicenter (China and Taiwan)	NR	22	22	Biopsy	23	18	NPI specimens from patients with no evidence of NPC, but with clinical symptoms that were compatible with NPC, were obtained from the same anatomical site. These biopsy samples were subsequently diagnosed as chronic inflammation or inflammation and necrosis	15	21												12 30 bp del 12 Xhol loss	Sequencing
Nicholls, 2004 [38]	China	NR	18	18	Biopsy	10	10	Total	18													8 30 bp del	
						5	5	Peripheral TCL										1				4 30 bp del 1 China1 1 Med-	Sequencing, monoclonal antibodies, peptide binding
						2	2	EBV+ HL														1 30 bp del, 1 Med-	
						3	3	Post-transplant lymphoproliferative disease														3 bp del, 3 China1	
Zhang, 2004 [39]	China	NR	117	99	Biopsy	53	46	Healthy donors PBMCs	87													4	Sequencing
Dardari, 2006 [40]	Morocco	NR	81	81	Total	30	14 PCR+	Healthy donors PBMCs	58			2										6 30 bp del 3 69 bp del	PCR and sequencing
			61	61	Biopsy				51			2											
			20	20	PBMCs				7			/											
Hadhri-Guiga, 2006 [41]	Tunis	NR	74	74	Total	20	17	Control patients with clinical symptoms indicative of NPC but whose postnasal biopsies were confirmed as histologically normal	43			2										9 30 bp del 0 69 bp del	Xhol digestion and sequencing
			42	42	Biopsy				28			2											
			32	32	PB lymphocytes				15			0											
See, 2008 [42]	Malaysia	NR	77	77	Total	10	8	Non-malignant nasopharyngeal tissue samples	26	45												0 30 bp del 0 Xhol loss	Xhol digestion and sequencing
			42	42	Biopsy				19	34													
			35	35	Plasma				7	11													
Tiwawech, 2008 [43]	Thailand	Case– control	75	75	PB leukocytes	44	44	Total	44					0	20	0	6	0	0	0	4	16	PCR and sequencing
						20	20	Randomly recruited age-matched (mean age ± 5 years) healthy subjects															
						24	24	Non-NPC patients with cancer and other disease															
Li, 2009 [44]	China	NR	150	150	Total	269	253	Total	74					1	131	3	11	0	0	0	3	87 30 bp del 91 China1 5 China2 30 B95-8 1 Med 12 other strains	PCR and sequencing
			50	50	Biopsy	15	9	Biopsy from patients with nasopharyngeal chronic inflammation	39					0	94	2	5	0	0	0	0	8 30 bp del 14 China1 1 China2	
			50	50	TW	9	9	TW from patients with nasopharyngeal chronic inflammation	30					1	37	1	6	0	0	0	3	6 30 bp del 8 China1	
			50	50	Serum	9	9	Blood from patients with nasopharyngeal chronic inflammation	0													0 30 bp del	
						55	55	TW from patients with other cancers														0 30 bp del 30 China1 20 B95-8 2 China2 1 Med 6 other strains	
						63	63	Biopsy samples from patients with other cancers														40 30 bp del	
						59	52	TW from healthy Cantonese donors														33 30 bp del 39 China1 10 B95-8 2 China2 6 other strains	
						59	56	PB from healthy Cantonese donors														0 30 bp del	
Banko, 2012 [45]	Serbia	NR	16	16	Biopsy	37	37	Total	1			3	0	5	1	6	0	0	0	4		12 30 bp del 1 69 bp del 1 27 bp del 12 B95-8 12 China1 7 Med	Sequencing
						30	30	Plasma samples from patients with mononucleosis syndrome														10 30 bp del 0 69 bp del 1 27 bp del 10 B95-8 10 China1 6 NC 4 Med	
						6	6	Plasma samples after renal transplantation														2 30 bp del 1 69 bp del 0 27 bp del 2 B95-8 2 China1 2 Med	
						1	1	HL biopsy														0 30 bp del 0 69 bp del 0 27 bp del 1 Med	
Gurtsevitch, 2013 [46]	Russia	NR	57	57	Total	69	55	Total	5			12		16	2	29	0	0	0	6	4	19 30 bp del 1 69 bp del 21 B95-8 16 China1 5 Med+ 6 Med- 6 NC 0 other	Sequencing
			21	21	Biopsy	20	14	OTOC (patients with cancer of the oral mucosa, tongue, sublingual tonsil, and some other malignant affections of the oral cavity) biopsy	3			6		6	2	10	0	0	0	1	2	OTOC biopsy 7 30 bp del 1 69 bp del 2 B95-8 5 China1 3 Med+ 2 Med- 2 NC 0 other	
			16	16	PB	20	13	OTOC blood	1			1		4	0	9	0	0	0	2	1	OTOC blood 8 30 bp del 0 69 bp del 2 B95-8 7 China1 1 Med+ 1 Med- 1 NC 0 other	
						20	19	Blood donors														Blood donors 15 B95-8 1 China1 0 Med+ 1 Med- 2 NC 0 other	
			20	20	Lavage	9	9	OTOC oropharyngeal lavage	1			5		6	0	10	0	0	0	3	1	OTOC lavage. 4 30 bp del 0 69 bp del 2 B95-8 3 China1 1 Med+ 2 Med- 1 NC 0 other	
Senyuta, 2014 [47]	Russia	NR	56	56	Total	54	54	Total	6			12		15	2	30	0	0	0	5	4	22 30 bp del 1 69 bp del 20 B95-8 17 China1 7 Med+ 6 Med- 7 NC 0 other	Sequencing
			22	22	Biopsy	14	14	Biopsy from patients with other (non-nasopharyngeal carcinoma) tumors of the oral cavity—cancers of the mucous membrane of the tongue (3), floor of the mouth (2), cheek (1), retro molar area (3), lower jaw (4), and palate (5)	4			6		6	2	11	0	0	0	1	2	Other ca biopsy 7 30 bp del 1 69 bp del 2 B95-8 5 China1 3 Med+ 2 Med- 2 NC 0 other	
			15	15	PB	12	12	Non-nasopharyngeal carcinoma blood samples	1			1		3	0	9	0	0	0	2	1	Blood other ca 9 30 bp del 0 69 bp del 1 B95-8 8 China1 1 Med+ 1 Med- 1 NC 0 other	
						19	19	Blood donors														Blood donors 1 30 bp del 0 69 bp del 15 B95-8 1 China1 0 Med+ 1 Med- 2 NC 0 other	
			19	19	TW	9	9	TW from other ca	1			5		6	0	10	0	0	0	2	1	TW other ca 4 30 bp del 0 69 bp del 2 B95-8 3 China1 1 Med+ 2 Med- 1 NC 0 other	
Neves, 2015 [48]	Portugal	Case– control	41	41	PB	43	43	PB from healthy controls	0													11	PCR
Karbalaie, 2019 [49]	Iran	Cross- sectional	7	7	Biopsy	3	3	Nasal, vocal cord and tongue ca	3													1	PCR

Abbreviations: NPC—nasopharyngeal carcinoma; TW—throat washing; PBMCs—peripheral blood mononuclear cells; PB—peripheral blood; PGC—parotid gland carcinoma; OTOC—other tumors of the same anatomical region; ca—carcinoma; BL—Burkitt`s lymphoma; non-HL—non-Hodgkin lymphoma; HL—Hodgkin lymphoma; OHL—oral hairy leucoplakia; TCL—T-cell lymphoma; BCL—B-cell lymphoma; IM—infectious mononucleosis; LELC-LG—lympho-epithelioma like carcinomas of lung; LELC-SG—lympho-epithelial carcinomas of salivary gland; SNCAs—sinonasal carcinomas; GACAs—gastric carcinomas; HBD—healthy blood donors; TNKLs—T/natural killer cell lymphoma; NPI—non-neoplastic counterparts.

## Data Availability

All detailed information about reasons for inclusion/exclusion and quality assessment, as well as Supplementary Materials are available at https://osf.io/znrwj/.

## Supplementary Materials

The following are available online at https://www.mdpi.com/article/10.3390/pathogens10081057/s1, Table S1: Summary of findings.
