# Supplementary material for: A Systematic Review of Epstein–Barr Virus Latent Membrane Protein 1 (LMP1) Gene Variants in Nasopharyngeal Carcinoma
Source: Pathogens. 2021 Aug 20;10(8):1057. doi: 10.3390/pathogens10081057 (PMC8401687; doi:10.3390/pathogens10081057)
Supplement: Supplementary file 1 [file pathogens-10-01057-s001.zip › pathogens-1302275-supplementary.pdf]

Table S1. Summary of findings.

Question: LMP1 gene variants in Nasopharyngeal carcinoma

Certainty assessment							№ of patients		Effect		Certainty	Importance
№ of studies	Study design	Risk of bias	Inconsistency	Indirectness	Imprecision	Other considerations	LMP1 variants		Relative (95% CI)	Absolute (95% CI)		
**XhoI loss in NPC vs. other tu biopsy**												
10	observational studies	not serious [a,b]	not serious	not serious	not serious	all plausible residual confounding would reduce the demonstrated effect	319/384 (83.1%)	102/190 (53.7%)	**OR 6.19** (3.55 to 10.78)	**341 more per 1,000** (from 268 more to 389 more)	⊕⊕⊕◯ MODERATE	CRITICAL
**XhoI loss NPC vs. healthy respondents biopsy**												
4	observational studies	serious [a,b]	not serious	not serious	serious [c]	strong association all plausible residual confounding would reduce the demonstrated effect	116/122 (95.1%)	45/73 (61.6%)	**OR 14.17** (4.99 to 40.20)	**341 more per 1,000** (from 273 more to 368 more)	⊕⊕◯◯ LOW	CRITICAL
**XhoI loss NPC biopsy vs. healthy TWs**												
4	observational studies	serious [a,b]	not serious	not serious	serious [c]	very strong association all plausible residual confounding would suggest spurious effect, while no effect was observed	147/154 (95.5%)	49/65 (75.4%)	**OR 24.60** (4.42 to 136.94)	**233 more per 1,000** (from 177 more to 244 more)	⊕⊕⊕◯ MODERATE	CRITICAL
**XhoI loss NPC vs. other EBV-associated non-NPC tumors biopsy in endemic regions**												
7	observational studies	serious [a,b]	not serious	not serious	not serious	all plausible residual confounding would reduce the demonstrated effect	215/244 (88.1%)	96/124 (77.4%)	**OR 2.10** (0.94 to 4.68)	**104 more per 1,000** (from 11 fewer to 167 more)	⊕⊕◯◯ LOW	CRITICAL
**XhoI loss NPC vs. other EBV-associated non-NPC tmorsu biopsy in non-endemic regions**												
3	observational studies	very serious [a,b,c]	not serious	not serious	serious [c]	very strong association all plausible residual confounding would reduce the demonstrated effect	9/25 (36.0%)	1/50 (2.0%)	**OR 11.84** (2.32 to 60.45)	**175 more per 1,000** (from 25 more to 532 more)	⊕⊕◯◯ LOW	CRITICAL

Certainty assessment							№ of patients		Effect		Certainty	Importance
№ of studies	Study design	Risk of bias	Inconsistency	Indirectness	Imprecision	Other considerations	LMP1 variants		Relative (95% CI)	Absolute (95% CI)		
**30-bp deletion NPC vs. healthy respondents biopsy**												
4	observational studies	not serious	not serious	not serious	not serious	all plausible residual confounding would reduce the demonstrated effect	98/128 (76.6%)	36/57 (63.2%)	**OR 3.53** (1.48 to 8.43)	**227 more per 1,000** (from 86 more to 304 more)	⨁⨁⨁◯ MODERATE	IMPORTANT
**30-bp deletion NPC biopsy vs. healthy respondents TWs**												
6	observational studies	not serious	not serious	not serious	not serious	all plausible residual confounding would reduce the demonstrated effect	248/273 (90.8%)	119/171 (69.6%)	**OR 3.77** (2.21 to 6.44)	**200 more per 1,000** (from 139 more to 241 more)	⨁⨁⨁◯ MODERATE	IMPORTANT
**30-bp deletion NPC biopsy vs. other EBV-associated non-NPC tumors biopsy**												
20	observational studies	not serious	serious	serious [d]	not serious	all plausible residual confounding would reduce the demonstrated effect	393/533 (73.7%)	246/441 (55.8%)	**OR 1.79** (0.79 to 4.04)	**135 more per 1,000** (from 59 fewer to 278 more)	⨁◯◯◯ VERY LOW	IMPORTANT
**30-bp deletion NPC vs. healthy respondents TWs**												
3	observational studies	not serious	not serious	not serious	not serious	all plausible residual confounding would reduce the demonstrated effect	79/153 (51.6%)	55/96 (57.3%)	**OR 1.25** (0.71 to 2.21)	**54 more per 1,000** (from 85 fewer to 175 more)	⨁⨁⨁◯ MODERATE	IMPORTANT
**30-bp deletion NPC blood vs Healthy blood**												
5	observational studies	not serious	serious [e]	serious [d]	not serious	all plausible residual confounding would reduce the demonstrated effect	52/201 (25.9%)	34/179 (19.0%)	**OR 0.82** (0.18 to 3.83)	**29 fewer per 1,000** (from 149 fewer to 283 more)	⨁◯◯◯ VERY LOW	IMPORTANT
**30-bp deletion NPC vs. other EBV-associated non-NPC tumors biopsy in endemic regions**												

Certainty assessment							№ of patients		Effect		Certainty	Importance
№ of studies	Study design	Risk of bias	Inconsistency	Indirectness	Imprecision	Other considerations	LMP1 variants		Relative (95% CI)	Absolute (95% CI)		
7	observational studies	not serious	not serious	not serious	not serious	all plausible residual confounding would reduce the demonstrated effect	197/223 (88.3%)	110/142 (77.5%)	**OR 1.59** (0.83 to 3.06)	**71 more per 1,000** (from 34 fewer to 139 more)	⨁⨁⨁◯ MODERATE	IMPORTANT
**30-bp deletion NPC vs. healthy respondents biopsy in endemic regions**												
5	observational studies	not serious	serious [e]	not serious	serious [c]	strong association all plausible residual confounding would reduce the demonstrated effect	135/171 (78.9%)	45/80 (56.3%)	**OR 6.91** (1.18 to 40.35)	**336 more per 1,000** (from 40 more to 419 more)	⨁⨁◯◯ LOW	IMPORTANT
**30-bp deletion NPC biopsy vs. healthy respondents TWs in endemic regions**												
6	observational studies	not serious	serious [c]	not serious	not serious	all plausible residual confounding would reduce the demonstrated effect	222/247 (89.9%)	114/155 (73.5%)	**OR 2.80** (1.62 to 4.84)	**151 more per 1,000** (from 83 more to 195 more)	⨁⨁◯◯ LOW	IMPORTANT
**30-bp deletion NPC vs. other EBV-associated non-NPC tumors biopsy in non-endemic regions**												
7	observational studies	not serious	not serious	serious [d]	not serious	all plausible residual confounding would reduce the demonstrated effect	20/84 (23.8%)	32/80 (40.0%)	**OR 0.67** (0.33 to 1.36)	**91 fewer per 1,000** (from 220 fewer to 76 more)	⨁⨁◯◯ LOW	IMPORTANT
**69-bp deletion NPC vs. other EBv-associated non-NPC tumors biopsy**												
4	observational studies	not serious	not serious	not serious	not serious	all plausible residual confounding would reduce the demonstrated effect	18/123 (14.6%)	5/62 (8.1%)	**OR 1.70** (0.63 to 4.61)	**49 more per 1,000** (from 28 fewer to 207 more)	⨁⨁⨁◯ MODERATE	IMPORTANT
**69-bp deletion NPC biopsy vs. healthy respondents periheral blood**												
4	observational studies	not serious	serious [e]	not serious	not serious	all plausible residual confounding would reduce the demonstrated effect	17/168 (10.1%)	5/106 (4.7%)	**OR 2.22** (0.26 to 18.60)	**52 more per 1,000** (from 34 fewer to 432 more)	⨁⨁◯◯ LOW	IMPORTANT

Certainty assessment							№ of patients		Effect		Certainty	Importance
№ of studies	Study design	Risk of bias	Inconsistency	Indirectness	Imprecision	Other considerations	LMP1 variants		Relative (95% CI)	Absolute (95% CI)		
**B95-8 NPC vs. healthy respondents biopsy**												
3	observational studies	serious [a]	serious [e]	serious [d]	not serious	all plausible residual confounding would reduce the demonstrated effect	12/62 (19.4%)	5/29 (17.2%)	**OR 1.27** (0.44 to 3.67)	**37 more per 1,000** (from 88 fewer to 261 more)	⊕◯◯◯ VERY LOW	NOT IMPORTANT
**B95.8-A NPC vs. healthy respondents TWs**												
3	observational studies	serious [a]	serious [e]	serious [d]	not serious	all plausible residual confounding would reduce the demonstrated effect	10/83 (12.0%)	14/29 (48.3%)	**OR 0.16** (0.00 to 5.90)	**353 fewer per 1,000** (from -- to 364 more)	⊕◯◯◯ VERY LOW	IMPORTANT
**B95.8/A NPC vs. healthy respondents peripheral blood**												
3	observational studies	not serious	not serious	not serious	not serious	all plausible residual confounding would suggest spurious effect, while no effect was observed	10/65 (15.4%)	46/66 (69.7%)	**OR 0.06** (0.02 to 0.17)	**576 fewer per 1,000** (from 653 fewer to 416 fewer)	⊕⊕⊕◯ MODERATE	IMPORTANT
**China 1 NPC vs. other EBV-associated non-NPC tumors biopsy**												
4	observational studies	not serious	not serious	not serious	not serious	all plausible residual confounding would suggest spurious effect, while no effect was observed	12/76 (15.8%)	13/36 (36.1%)	**OR 0.33** (0.13 to 0.85)	**204 fewer per 1,000** (from 293 fewer to 37 fewer)	⊕⊕⊕◯ MODERATE	IMPORTANT
**China 1 NPC vs. other EBV-associated non-NPC tumors peripheral blood**												
3	observational studies	not serious	serious [e]	not serious	not serious	all plausible residual confounding would suggest spurious effect, while no effect was observed	20/61 (32.8%)	24/39 (61.5%)	**OR 0.10** (0.00 to 2.34)	**477 fewer per 1,000** (from -- to 174 more)	⊕⊕◯◯ LOW	NOT IMPORTANT
**China 1 NPC vs. other EBV-associated non-NPC tumors TWs**												

Certainty assessment							№ of patients		Effect		Certainty	Importance
№ of studies	Study design	Risk of bias	Inconsistency	Indirectness	Imprecision	Other considerations	LMP1 variants		Relative (95% CI)	Absolute (95% CI)		
3	observational studies	not serious	serious [e]	serious [d]	serious [c]	all plausible residual confounding would suggest spurious effect, while no effect was observed	37/87 (42.5%)	36/77 (46.8%)	**OR 0.25** (0.01 to 7.88)	**288 fewer per 1,000** (from 459 fewer to 406 more)	⊕◯◯◯ VERY LOW	NOT IMPORTANT
**Med NPC vs. other EBv-associated non-NPC tumours biopsy**												
4	observational studies	not serious	not serious	not serious	not serious	all plausible residual confounding would suggest spurious effect, while no effect was observed	29/76 (38.2%)	13/36 (36.1%)	**OR 1.14** (0.50 to 2.63)	**31 more per 1,000** (from 141 fewer to 237 more)	⊕⊕⊕◯ MODERATE	IMPORTANT
**Med NPC vs. other EBV-associated non-NPC tumors peripheral blood**												
3	observational studies	not serious	serious [e]	serious [d]	not serious	all plausible residual confounding would suggest spurious effect, while no effect was observed	18/61 (29.5%)	6/39 (15.4%)	**OR 2.26** (0.21 to 24.18)	**137 more per 1,000** (from 117 fewer to 661 more)	⊕◯◯◯ VERY LOW	NOT IMPORTANT
**Med NPC vs. other EBV-associated non-NPC tumors TWs**												
3	observational studies	not serious	not serious	not serious	not serious	all plausible residual confounding would suggest spurious effect, while no effect was observed	21/87 (24.1%)	7/77 (9.1%)	**OR 1.95** (0.67 to 5.69)	**72 more per 1,000** (from 28 fewer to 272 more)	⊕⊕⊕◯ MODERATE	IMPORTANT
**North Carolina NPC vs. other EBV-associated non-NPC tumors biopsy**												
3	observational studies	not serious	not serious	not serious	not serious	all plausible residual confounding would reduce the demonstrated effect	2/59 (3.4%)	6/35 (17.1%)	**OR 0.20** (0.04 to 0.90)	**132 fewer per 1,000** (from 163 fewer to 14 fewer)	⊕⊕⊕◯ MODERATE	IMPORTANT

CI: Confidence interval; **OR:** Odds ratio; TW: throat washings

Explanations

a. There was no confounding control in the study design, neither in the analysis.

b. There was a selection bias due to inappropriately classified respondents (by regions, not by outcome).

c. 95%CI of the odds ratio is wide, as thresholds for grading were considered trivial from 1 to 2.5, small from 2.5 to 5, moderate from 5 to 10, and large 10 and more

d. There were results in both directions i.e. some studies reported higher, other lower prevalence of LMP1 gene variant in NPCs

e. There was a great heterogeneity between studies assessed by I2 statistics in Review Manager
